# Supplementary material for: Biological Activity of Optimized Codon Bovine Type III Interferon Expressed in Pichia pastoris
Source: Viruses. 2023 Apr 30;15(5):1101. doi: 10.3390/v15051101 (PMC10221290; doi:10.3390/v15051101)
Supplement: Supplementary file 1 [file viruses-15-01101-s001.zip › viruses-2373777-supplementary.pdf]

Supplement Figure legends

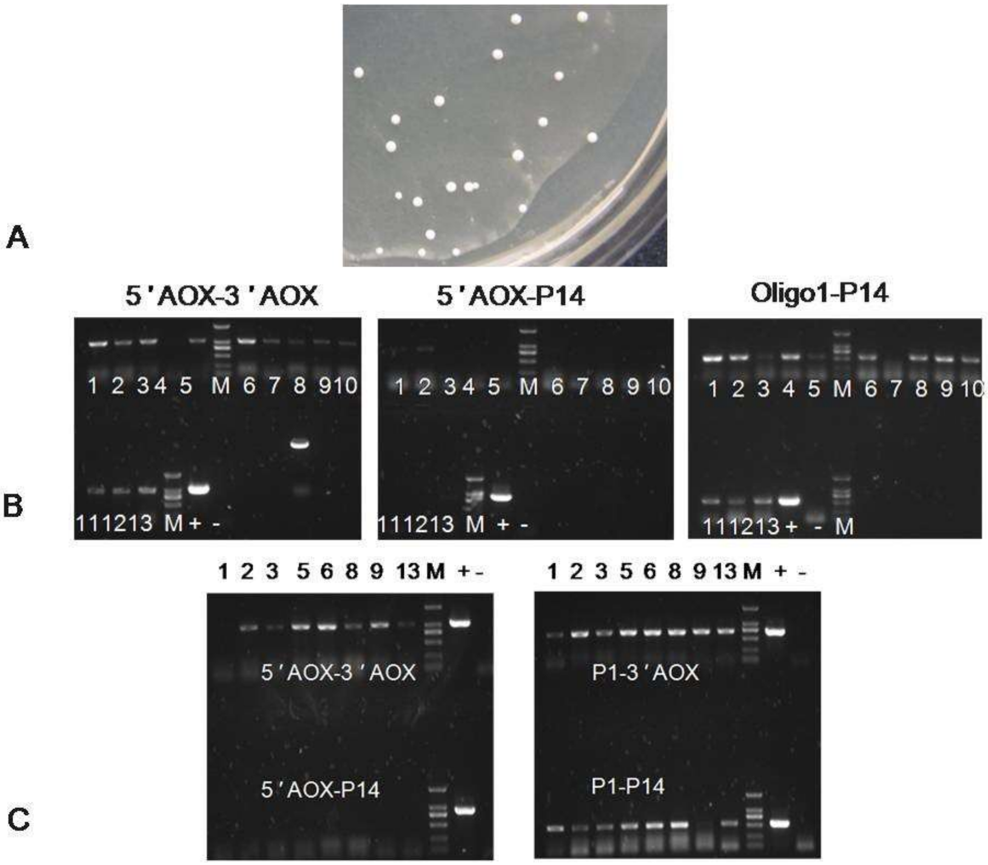

Figure. S1. The selection and identification of recombinant GS115-pPICZαA-boIFN-λ3

(A). The colony of the GS115-pPICZαA-boIFN-λ3. (B). PCR product amplified from the first generation of GS115-pPICZαA-boIFN-λ3 1to13; + Positive control; - Negative control. (C). PCR identified from the second generation of GS115-pPICZαA-boIFN-λ3 1, 2, 3, 5, 6, 8, 9 and 13; + Positive control; - Negative control.

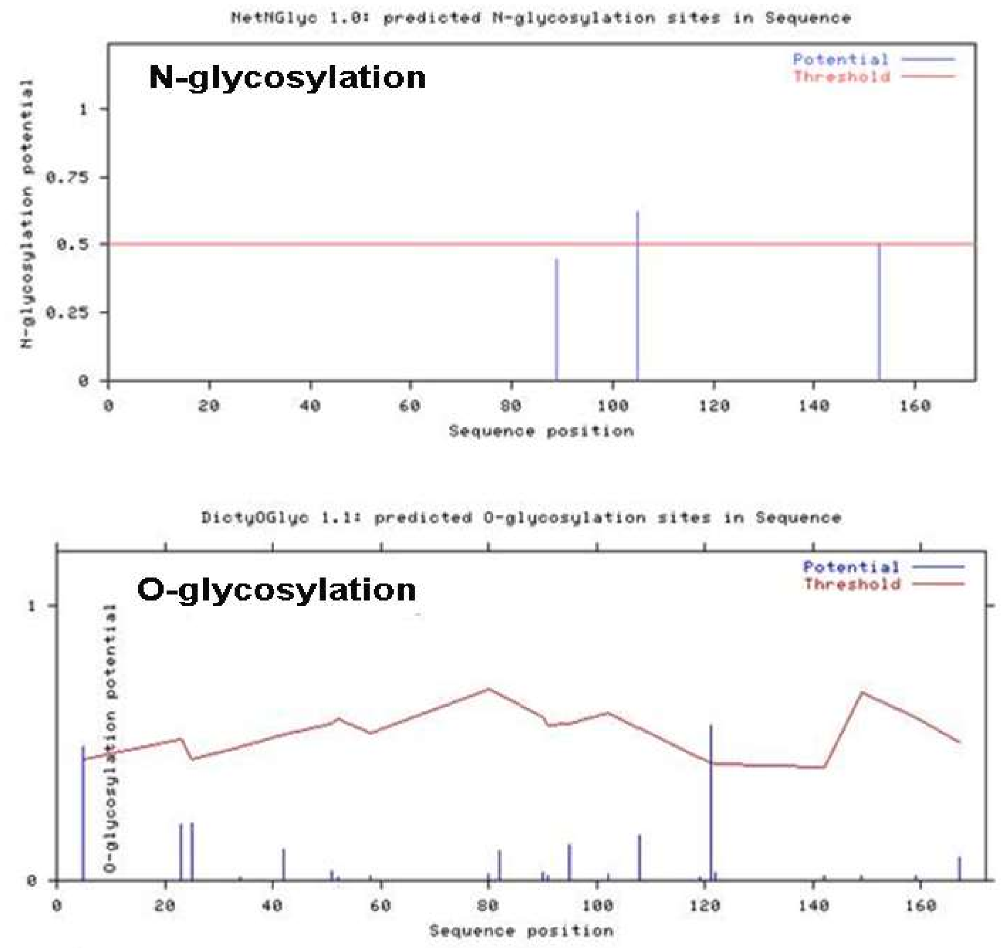

Figure. S2. The predicted N-linked and O-linked glycosylation sequence of boIFN-λ3

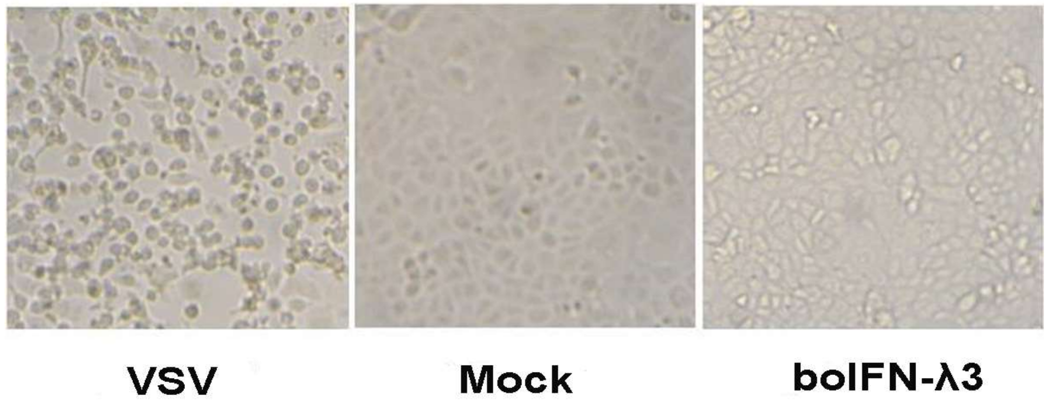

Figure. S3. Inhibation of VSV in MDBK cell by recombinant boIFN-λ3
